# Occult RV systolic dysfunction detected by CMR derived RV circumferential strain in patients with pectus excavatum

**DOI:** 10.1371/journal.pone.0189128

**Published:** 2017-12-11

**Authors:** Vien T. Truong, Candice Y. Li, Rebeccah L. Brown, Ryan A. Moore, Victor F. Garcia, Eric J. Crotty, Michael D. Taylor, Tam M. N. Ngo, Wojciech Mazur

**Affiliations:** 1 Department of Cardiology, The Christ Hospital; Cincinnati, Ohio, United States of America; 2 Department of Cardiology, Pham Ngoc Thach University of Medicine, Ho Chi Minh City, Viet Nam; 3 Department of Cardiology, Cincinnati Children’s Hospital Medical Center, Cincinnati, Ohio, United States of America; 4 Department of Surgery, Cincinnati Children’s Hospital Medical Center, Cincinnati, Ohio, United States of America; 5 Department of Radiology, Cincinnati Children’s Hospital Medical Center, Cincinnati, Ohio, United States of America; Ospedale del Cuore G Pasquinucci Fondazione Toscana Gabriele Monasterio di Massa, ITALY

## Abstract

**Aims:**

To investigate the right ventricular (RV) strain in pectus excavatum (PE) patients using cardiac magnetic resonance tissue tracking (CMR TT).

**Materials and methods:**

Fifty consecutive pectus excavatum patients, 10 to 32 years of age (mean age 15 ± 4 years), underwent routine cardiac magnetic resonance imaging (CMR) including standard measures of chest geometry and cardiac size and function. The control group consisted of 20 healthy patients with a mean age of 17 ± 5 years. RV longitudinal and circumferential strain magnitude was assessed by a dedicated RV tissue tracking software.

**Results:**

Fifty patients with images of sufficient quality were included in the analysis. The mean right and left ventricular ejection fractions were 55 ± 5% and 59 ± 4%. The RV global longitudinal strain was -21.88 ± 4.63%. The RV circumferential strain at base, mid-cavity and apex were -13.66 ± 3.09%, -11.31 ± 2.79%, -20.73 ± 3.45%, respectively. There was no statistically significant decrease in right ventricular or left ventricular ejection fraction between patients and controls (p > 0.05 for each). There was no significant difference in RV global longitudinal strain between two groups (-21.88 ± 4.63 versus -21.99 ± 3.58; *p* = 0.93). However, there was significant decrease in mid-cavity circumferential strain magnitude in pectus patients compared with controls (-11.31 ± 2.79 versus -16.19 ± 2.86; *p* < 0.001). PE patients had a significantly higher basal circumferential strain (-13.66 ± 3.09% versus -9.76 ± 1.79; *p* < 0.001) as well as apical circumferential strain (-20.73 ± 3.45% versus -12.07 ± 3.38) than control group.

**Conclusion:**

Mid-cavity circumferential strain but not longitudinal strain is reduced in pectus excavatum patients. Basal circumferential strain as well as apical circumferential strain were increased as compensatory mechanism for reduced mid-cavity circumferential strain. Further studies are needed to establish clinical significance of this finding.

## Introduction

Pectus excavatum (PE) is the most common congenital deformity of the anterior chest wall that affects both appearance and function [1]. Pectus excavatum occurs in 1 in 300–400 births with a male predominance [1, 2]. The degree of abnormal chest wall deformity determines its functional effect, particularly its cardiopulmonary impairment and physiologic limitations [2]. Patient may be completely asymptomatic or presents with exertional dyspnea, decreased endurance, tachycardia, and palpitations [2, 3]. The cause of PE remains unknown. Impairment in the growth of the sternum and biomechanical properties of costal cartilage, are proposed in the pathogenesis [4]. Many patients with PE have associated alterations in right ventricular (RV) morphology and function and cardiopulmonary disturbance may contribute to symptoms in patients with PE [5–7]. Guidelines for the assessment of cardiovascular function remain undefined.

A non-contrast computerized tomographic (CT) scan is helpful to examine the deformity of the bony and cartilaginous skeleton, which clearly finds any cardiac compression or displacement. Assessing RV function using echocardiography is limited by the complex RV geometry, retrosternal position, and complex motion [8], especially in pectus excavatum due to deformity of the anterior chest wall. In contrast, MRI provides highly reproducible information on RV myocardial motion and function and can be used instead of CT scan to reduce radiation exposure in pectus excavatum. Cardiac magnetic resonance (CMR) imaging has emerged as validated tool in patients with pectus excavatum [6, 9, 10]. The purpose of our study is to investigate the RV strain in pectus excavatum patients using cardiac magnetic resonance tissue tracking (CMR TT).

## Materials and methods

### Patient population

Fifty pectus excavatum patients without known RV pathology, 10 to 32 years of age (mean age 15 ± 4 years), underwent cardiac magnetic resonance imaging (CMR) as part of standard clinical evaluation. The control group consisted of 20 healthy patients with a mean age of 17 ± 5 years. The control group was referred for the following suspected conditions: atypical chest pain, suspected coronary artery anomaly, cardiac mass by echocardiography (typically prominent moderator band). We excluded not only patients with reduced LVEF, segmental wall motion abnormalities but also abnormal longitudinal and circumferential strain. Pectus excavatum patients or the control group with known conditions affecting RV function such as congenital heart disease, cardiomyopathy, valvular heart disease, sleep apnea, morbid obesity, known or suspected pulmonary hypertension, were excluded from the study. This study was approved by the institutional review board (IRB) of Cincinnati Children’ Hospital Medical Center.

### CMR acquisition

Standard CMR acquisition was performed as reported previously [11]. Briefly, imaging was performed on a 1.5 Tesla scanner (Ingenia, Philips Healthcare; Best, Netherlands), using a phased-array coil. A horizontal long-axis image and a short-axis RV stack from the atrioventricular ring to the RV apex were acquired using an SSFP pulse sequence (repetition time of 3.2 ms; echo time of 1.7 ms; flip angle of 60°; sequential 7 mm slices with no interslice gap). There were 30 phases per cardiac cycle resulting in a mean temporal resolution of 30–40 ms.

**Fig 1:**
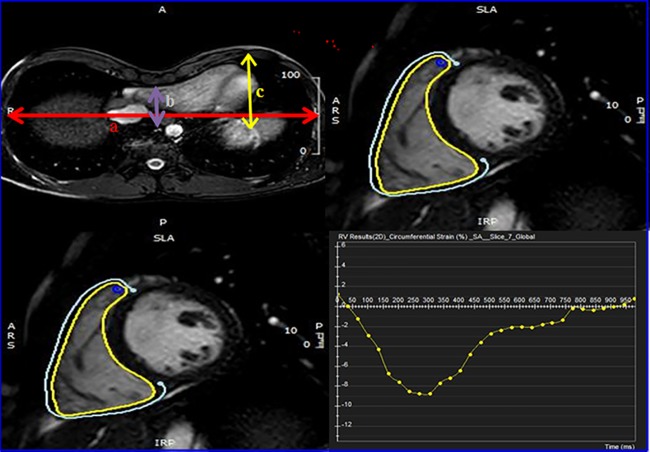
Pectus index and CMR tissue tracking. (A) The Haller Index (HI) is a ratio of the transverse diameter of the chest (line a) to the distance between the posterior aspect of the sternum and the anterior portion of the vertebra (line b): HI = a/b. The correction index (CI) measures the depression of the sternum relative to the anterior chest: CI = [(c-b)/c] x 100. (B) Right ventricular longitudinal strain. (C) and (D) Mid-cavity circumferential strain and peak value was recorded. The yellow colored contours show the tracking of the ventricle.

RV longitudinal and circumferential strain magnitude was assessed by a dedicated RV tissue tracking software [12]. The following clinical variables were collected for each patients: age, sex, body mass index (BMI- kg/m2), body surface area (BSA-m2), and heart rate (bpm). The cardiac and chest MR variables were: LV and RV end—diastolic volume (EDV-ml), end—systolic volume (ESV-ml), stroke volume (ml) as well as indexed (BSA) values were calculated, ejection fraction (EF-%), left ventricular mass index (g/m2), cardiac output (L/min), cardiac index (L/min/m2), Haller index, correction index, and depression index. The Haller index is a ratio of the transverse diameter of the chest to the anterior-posterior diameter, measured from the inner aspect of the sternum to the anterior aspect of the vertebral body at the level of greatest sternal depression (Fig 1)[13]. Correction index and depression index were evaluated according to methods previously described [14, 15].

### Tissue tracking

The RV myocardial deformation was quantified using a prototype of RV specific CVI42 Tissue Tracking software (Circle Cardiovascular Imaging, Calgary, Canada). First, an experienced operator traced the RV endocardial and epicardial borders at the end diastolic (ED) phase in both short-axis and long-axis cine images. The software then constructed a 3D deformable myocardial model based on the tracing, assuming the myocardium is nearly incompressible [16]. In each of the subsequent frames the displacements of the myocardial tissues, including the borders were determined using a gradient-based optical flow method with an incompressible model constraint. The propagated myocardial tissue across the cardiac cycle was verified by the operator to ensure the accuracy of the propagation. Strain values (along the longitudinal, circumferential, and radial directions) for each tissue point as well as the global strain values for the short-axis and long-axis views were automatically derived by the software [12]. The right ventricle was divided into basal, mid-cavity, and apical segments to derive regional deformation parameters.

### Strain analysis

The horizontal long axis was used for calculation of longitudinal strain, while the short axis at the level of greatest sternal depression was used to calculate mid-cavity circumferential strain of the right ventricle. The apical and basal circumferential strain were calculated from the apical and basal short-axis views respectively. The interventricular septum was not included in the strain calculation. Endocardial and epicardial contours were drawn in the cardiac phase with the most distinct myocardium boundaries; RV trabeculations were carefully excluded. The software automatically propagated contours throughout all phases. Longitudinal and mid-cavity circumferential strain were computed as shown in Fig 1 [12].

### Statistical analysis

Continuous variables are expressed as mean **±** standard deviation (SD) for normal distributions and median + interquartile range for non-normal distributions. Normality was tested using the Shapiro–Wilk test. For the evaluation of qualitative variables, we used the Chi-Square test. To test for significant differences between continuous variables in two groups, independent sample t-tests were performed for normally distributed variables and Mann-Whitney U test was performed for variables with non-normal distribution. Spearman's Rho correlation test was performed to evaluate the relationships among continuous variables. Statistical analysis was performed using the SPSS 22 software program (SPSS Inc., Chicago, IL, USA). A *P* value of < 0.05 was considered statistically significant.

## Results

Demographic, and CMR-derived parameters for the whole sample are shown in Table 1 and S1 File. The mean age of patients was the 15 ± 4 (years). There were 39 men with a mean age of 16 ± 4 years and 11 women with a mean age of 14 ± 4 years (*P* = 0.12). The mean value of LVEF and RVEF were 58.97 ± 4.07; 55.06 ± 4.94, respectively (Table 1). The mean Haller index was 5.71 ± 2.93 with a median index of 4.68 (4.05 to 6.78) (Table 2). There were 46 patients with severe chest wall phenotype, defined as a Haller index over 3.25. The LV global longitudinal strain and was -21.88 ± 4.63%. The RV circumferential strain at base, mid-cavity and apex were -13.66 ± 3.09%, -11.31 ± 2.79%, -20.73 ± 3.45%, respectively. Differences were not statistically significant for age, right ventricular or left ventricular ejection fraction (*p* > 0.05 for each). There was no significant difference in RV global longitudinal between patients and controls (-21.88 ± 4.63% versus -21.99 ± 3.58%; *p* = 0.93). However, there was significant decrease in mid-cavity circumferential strain magnitude in pectus patients compared with controls (-11.31 ± 2.79 versus -16.19 ± 2.86; p < 0.001) (Table 3). PE patients had a significantly higher basal circumferential strain (-13.66 ± 3.09% versus -9.76 ± 1.79; p < 0.001) as well as apical circumferential strain (-20.73 ± 3.45% versus -12.07 ± 3.38) than control group.

**Table 1:** Baseline characteristics of fifty pectus excavatum patients.

Age (years)	15 ± 4
Sex (male)	39 (78%)
Heart rate (bpm)	77 ± 18
BSA (m2)	1.61 ± 0.25
BMI (kg/m^2^)	18.8 ± 2.8
Left Ventricle	
Absolute	
EF (%)	59 ± 4
LVEDV (ml)	142 ± 34
LVESV (ml)	59 ± 17
SV (ml)	83 ± 19
CO (L/min)	6.2 ± 1.5
Normalized (BSA)	
EDV (ml/m^2^)	88 ± 13
ESV (ml/m^2^)	36 ± 7
SV (ml/m^2^)	52 ± 7
CI (L/min/m2)	3.9 ± 0.8
Mass (gm/m2)	45 ± 10
Right Ventricle	
Absolute	
EF (%)	55 ± 5
RVEDV (ml)	153 ± 41
RVESV (ml)	70 ± 23
SV (ml)	83 ± 19
CO (L/min)	6.2 ± 1.5
Normalized (BSA)	
EDV (ml/m^2^)	94 ± 16
ESV (ml/m^2^)	43 ± 10
SV (ml/m^2^)	52 ± 8
CI (L/min/m2)	3.9 ± 0.8

Continuous variables are expressed as mean ± standard deviation. Categorical variables are presented as n (%)

BSA: body surface area; EF: ejection fraction; EDV: end-diastolic volume; ESV: end-systolic volume; SV: stroke volume; CO: cardiac output; CI: cardiac index

**Table 2:** Chest index.

Haller index	4.68 (4.05 to 6.78)
Correction index	40.85 (26.47 to 78.90)
Depression index	0.67 ± 0.35

Normally distributed continuous variables are presented as mean ± standard deviation. Non-normally distributed continuous variables are presented as median (inter-quartile range).

**Table 3:** Basic demographics and patients characteristics between two groups.

	Normal (n = 20)	Pectus patient (n = 50)	P
Age (years)	17 ± 5	15 ± 4	0.12
RVEF (%)	57± 4	55 ± 5	0.12
LVEF (%)	58 ± 3	59 ± 4	0.46
RV longitudinal strain (%)	-21.99 ± 3.58	-21.88 ± 4.63	0.93
RV circumferential strain (%)			
Basal	-9.76 ± 1.79	-13.66 ± 3.09	< 0.001
Mid-cavity	-16.19 ± 2.86	-11.31 ± 2.79	< 0.001
Apex	-12.07 ± 3.38	-20.73 ± 3.45	< 0.001

Continuous variables are expressed as mean ± standard deviation.

**Fig 2:**
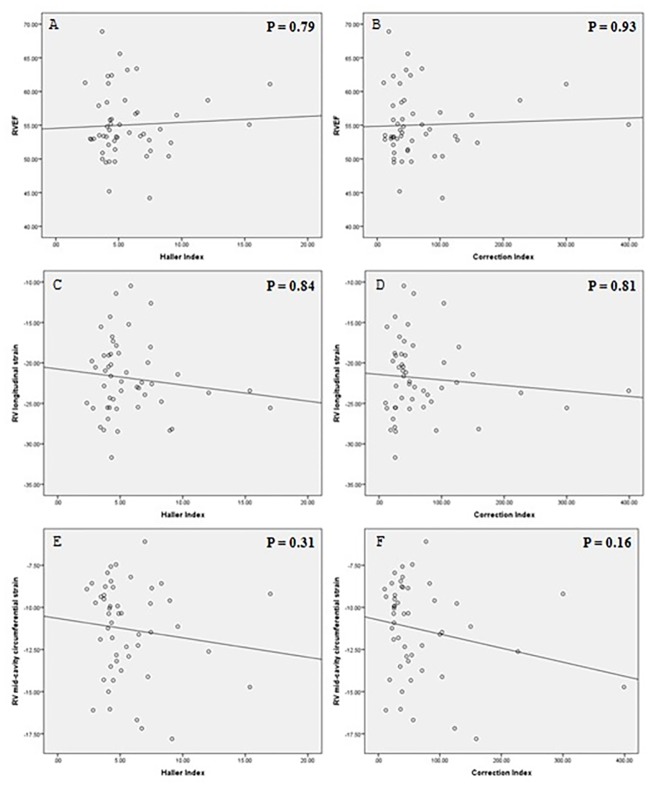
Relationship between pectus severity index and right ventricular ejection fraction, myocardial strain.

There were no correlations between RVEF and Haller index (Spearman’s = 0.039, *p* = 0.79), RV longitudinal strain and Haller index (Spearman’s = -0.029, *p* = 0.84), RV mid-cavity circumferential strain and Haller index (Spearman’s = -0.148, *P* = 0.31), RVEF and correction index (Spearman’s = 0.013, *p* = 0.93), RV longitudinal strain and correction index (Spearman’s = 0.036, *p* = 0.81), RV mid-cavity circumferential strain and correction index (Spearman’s = -0.202, *p* = 0.16) (Fig 2).

## Discussion

CMR TT has emerged as a useful technique for assessing RV myocardial function [12, 17]. This study assessed RV myocardial strain using CMR TT in a cohort of 50 patients with pectus excavatum. Our study showed no significant difference in RV longitudinal strain between the patients and normal controls. Patients with pectus excavatum had trend towards lower RVEF compared to the healthy control group, but it did not reach statistical significance. However, mid-cavity circumferential strain magnitude is significantly decreased in patient group. This may be explained by geometric distortion of the RV due to sternal compression in pectus excavatum. Saleh et al studied 30 patients with pectus excavatum with age ranges 14–67 years who underwent CMR. They found decreased RV ejection fraction (53.9 ± 9.6 versus 60.5 ± 9.5; *p* = 0.013), reduced right ventricular short axis dimensions (*p* < 0.05) both at end diastole and systole, and increased right ventricular long axis dimensions at end diastole (*p* < 0.05) compared to controls [10]. Töpper et al performed CMR in 38 patients (mean age 21 ± 8.3; 31 men) before and after surgical correction and found RVEF was decreased before surgery and improved significantly after successful correction (45.7% vs 48.3%, *P* = 0.0004) [9]. RVEF is significantly decreased in study of Saleh et al and Töpper et al but not in our study. This can be explained by severity of pectus excavatum. In our study, the Haller index was 5.71 ± 2.93 lower than those of Saleh et al (9.3 ± 5.0) and Töpper et al (9.64 ± 0.75). Furthermore, our study found that patients with PE had the higher basal and apical circumferential strain which may be explained as compensatory mechanism for reduced mid-cavity circumferential strain. Severeal recent studies demonstrated,in conditions leading to RV pressure or volume overload such as tetralogy of Fallot, pulmonary hypertension, or Systemic RV, the contractile pattern changes from longitudinal to circumferential shortening [18–20]. This might be the reason why longituidinal strain was not increased in our study. In addition, the RV with deep longitudinal myocardial layers that are aligned base to apex are likely to affect RV strain [21], which is also supported by the fact that a significant base-to-apex segmental strain gradient with much higher apical circumferential strain value in PE.

Recent studies demonstrate that for evaluating ventricle dysfunction, myocardial strain value can be used as a highly sensitive marker as opposed to the ejection fraction, since a reduction in the strain often precedes a decline in EF [22, 23]. De Siqueira et al retrospectively enrolled 116 patients (age 52.2 ± 12 years, 73.6% women) referred to CMR for pulmonary hypertension evaluation who underwent right heart catheterization. The author found the all strain values (longitudinal strain and circumferential strain) were significantly decreased in patients with pulmonary hypertension with normal RVEF compared to control group [23]. We did not find correlations between RVEF, RV longitudinal strain and RV circumferential strain with Haller index and correction index. Saleh et al also found no significant correlation between the RVEF and pectus severity index (r = -0.2061, *p* = 0.2746) [10]. The author also explain the possible reasons that included chest reconfiguration and cardiac left lateral shift [10]. These factors could be compensatory mechanisms to avoid the impairment in RV systolic function. Sigalet found the similar result that pectus index has not been shown to have any correlation with stroke volume [24].

## Limitations

Limitations of our study include its retrospective nature leading to possible selection bias. We were unable to report segmental longitudinal strain as well as radial strain due to poor reproducibility which is known as current software limitation. Moreover, the measured strain will be slightly different than the actual strain that would be measured by tracking the same myocardium without through plane motion. There is no easy way to fix this limitation using the 2-d system that we employ for strain measurement. The only true way to correct for this is using a 3-d cine acquisition to get an isotropic 3-d data set combined with a 3-d strain analysis. While that has been shown to work in limited research studies, the technique suffers from significantly reduced spatial and temporal resolution. This limitation affects equally PE and control patients, as such it is unlikely that it would affect findings of our study.

## Conclusion

Mid-cavity circumferential strain but not longitudinal strain is reduced in pectus excavatum patients and is more sensitive marked for occult RV dysfunction than RVEF. There was a compensatory increase in basal as well as apical circumferential strain representing RV adaptation to maintain systolic RV function without simultaneous increase in RV longitudinal strain. Further studies are needed to establish clinical significance of this finding.

## Supporting information

S1 File Datasheet for pectus excavatum study. (SAV)
